# The role of TNC in atherosclerosis and drug development opportunities

**DOI:** 10.7150/ijbs.89890

**Published:** 2024-01-01

**Authors:** Wujun Chen, Yanhong Wang, Chunling Ren, Sha Yu, Chao Wang, Jiyao Xing, Jiazhen Xu, Saisai Yan, Tingting Zhang, Qian Li, Xiaojin Peng, Yingchun Shao, Renshuai Zhang, Daijun Zhang, Dongming Xing

**Affiliations:** 1 Cancer Institute, The Affiliated Hospital of Qingdao University, Qingdao University, Qingdao Cancer Institute, Qingdao, Shandong, 266071, China.; 2 Department of Pharmacy, Women's and Children's Hospital Afliated to Qingdao University, Qingdao Women's and Children's Hospital, Qingdao, Shandong, 266000, China.; 3 Obstetrical Department, The Affiliated Hospital of Qingdao University, Qingdao, Shandong, 266003, China; 4 Qingdao Medical College, Qingdao University, Qingdao, Shandong, 266071, China.; 5 School of Life Sciences, Tsinghua University, Beijing, 100084, China.

**Keywords:** TNC, atherosclerosis, G11-iRGD, PL1, F16, ATN-RNA, drug development

## Abstract

Tenascin C (TNC), a rich glycoprotein of the extracellular matrix, exhibits a pro-atherosclerosis or anti-atherosclerosis effect depending on its location. TNC, especially its C domain/isoform (TNC-C), is strongly overexpressed in atherosclerotic plaque active areas but virtually undetectable in most normal adult tissues, suggesting that TNC is a promising delivery vector target for atherosclerosis-targeted drugs. Many delivery vectors were investigated by recognizing TNC-C, including G11, G11-iRGD, TN11, PL1, and PL3. F16 and FNLM were also investigated by recognizing TNC-A1 and TNC, respectively. Notably, iRGD was undergoing clinical trials. PL1 not only recognizes TNC-C but also the extra domain-B (EDB) of fibronectin (FN), which is also a promising delivery vector for atherosclerosis-targeted drugs, and several conjugate agents are undergoing clinical trials. The F16-conjugate agent F16IL2 is undergoing clinical trials. Therefore, G11-iRGD, PL1, and F16 have great development value. Furthermore, ATN-RNA and IMA950 were investigated in clinical trials as therapeutic drugs and vaccines by targeting TNC, respectively. Therefore, targeting TNC could greatly improve the success rate of atherosclerosis-targeted drugs and/or specific drug development. This review discussed the role of TNC in atherosclerosis, atherosclerosis-targeted drug delivery vectors, and agent development to provide knowledge for drug development targeting TNC.

## 1. Introduction

Coronary heart disease (CHD) is the leading cause of death worldwide and is primarily caused by atherosclerosis. Atherosclerotic plaque growth blocks blood flow, leading to ischemia of the surrounding tissue by promoting narrowing of the vessel lumen. Plaque ruptures lead to life-threatening myocardial infarction (MI), coronary artery disease (CAD), cerebral infarction (ischemic stroke), and acute coronary syndrome (ACS) by promoting platelet aggregation, fluid coagulation, and thrombosis. Atherosclerosis is characterized by the accumulation of lipids in the artery walls, accompanied by infiltration of immune cells and chronic inflammation. The early stage of atherosclerosis is the formation of "fatty streaks" that consist of cholesterol-laden foam cells. As the disease progresses, necrotic cells accumulate in the plaque to form necrotic nuclei that infiltrate numerous inflammatory cells and release proinflammatory cytokines and chemokines. Therefore, lipid-lowering, anti-inflammatory, or immunomodulatory therapy is necessary to delay the progression of atherosclerosis 1-3. However, atherosclerosis mainly occurs in the heart aorta. Many drugs have many side effects. These include statin-induced liver and neuromuscular toxicity, cramps, myalgia, necrotizing myopathy, and rhabdomyolysis 4, 5. Ezetimibe has a placebo-like side effect profile. PCSK9 inhibitors induce nasopharyngitis 6, 7. Therefore, the development of drugs that target aortic plaques without affecting other tissues will greatly improve the efficacy and reduce the side effects of drugs. Conjugative drugs that conjugate the genes highly expressed in pathological tissue but rarely expressed in normal tissue have a specificity and potency that traditional drugs cannot achieve. Current conjugative drugs include antibody‒drug conjugates (ADCs), antibody degraducer conjugates (ADeCs), aptamer-drug conjugates (ApDCs), antibody-oligonucleotide conjugates (AOCs), antibody fragment-drug conjugates (FDCs), immune-stimulating antibody conjugates (ISACs), radionuclide-drug conjugates (RDCs), small molecule-drug conjugates (SMDCs), and virus-like drug conjugates (VDCs). ADCs, RDCs, SMDCs, and ISACs are the most successful coupling drugs because many related drugs have been approved on the market or entered into clinical trials 8-10. Therefore, the development of these conjugative agents will greatly improve the success rate of drug development.

**Figure 1:**
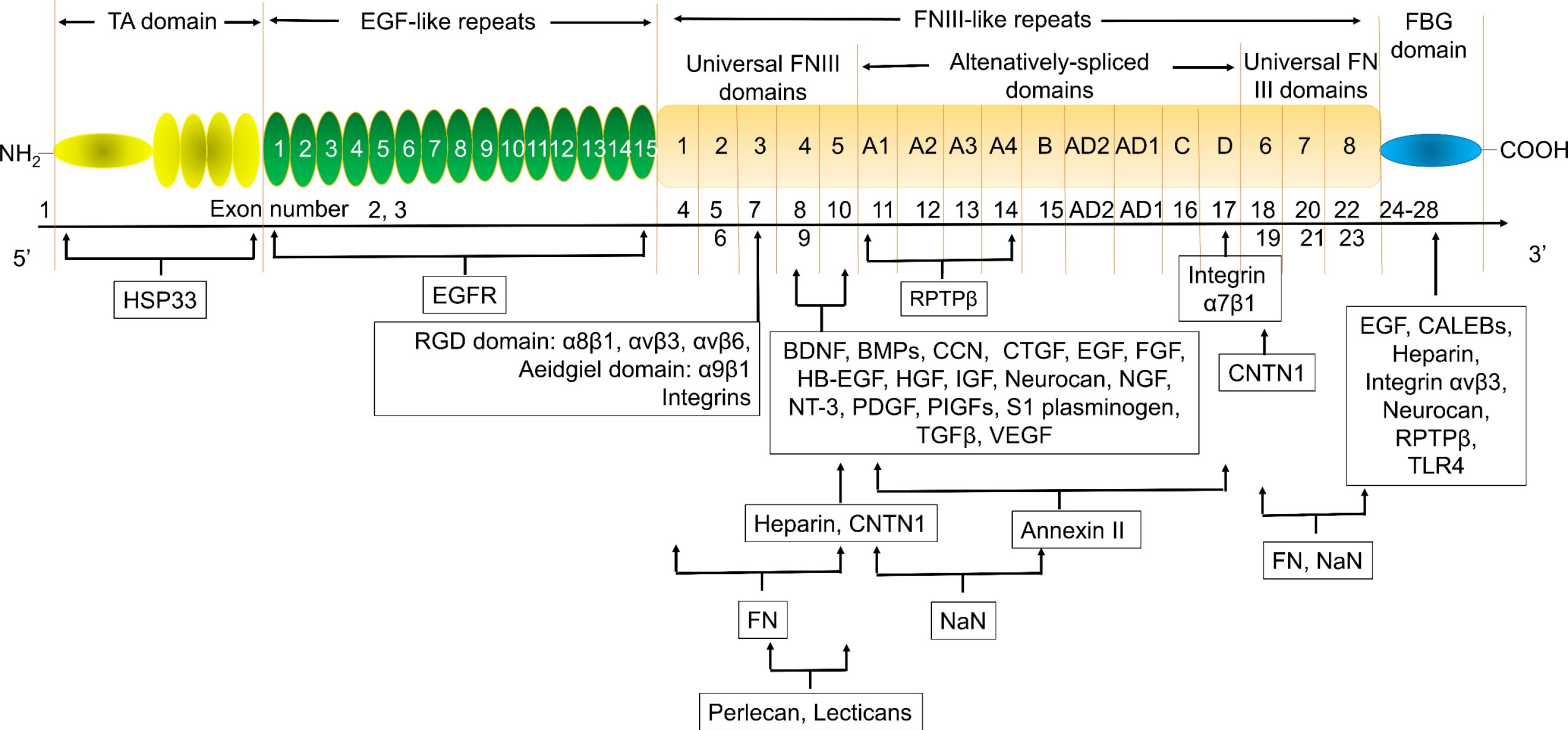
** The exon structure, corresponding domains, and binding factors of TNC.** BDNF, brain derived neurotrophic factor; BMPs, Bone morphogenetic proteins; CALEB, chicken acidic leucine-rich EGF-like domain containing brain protein; CNTN1, Contactin-1; CTGF, connective tissue growth factor; EGF, Epidermal growth factor; EGFR, EGF receptor; FBG, Fibrinogen-like globe; FN, Fibronectin; FNIII, Fibronectin type III; HB-EGF, heparin-binding EGF-like growth factor; FGF, fibroblast growth factor; HGF, hepatocyte growth factor; HSP33, heat shock protein 33; IGF, insulin-like growth factor; NaN, sodium channel subunit β2; NGF, Nerve growth factor; NT-3, Neurotrophin-3; PDGF, Platelet-derived growth factor; PIGF, placental growth factor; RPTPβ, receptor protein tyrosine phosphatase β; TA, Tenascin assembly; TGFβ, Transforming growth factor β; TLR4, toll-like receptor-4; VEGF, vascular endothelial growth factor. The information taken and modified from Refs 11-22.

Tenascin C (TNC) is a member of the extracellular matrix (ECM) protein family and plays a key role in wound healing and tissue remodeling. TNC has multiple biological roles by binding to and interacting with multiple genes. Heat shock protein 33 (HSP33) is responsible for TNC accumulation in cells. The epidermal growth factor (EGF)-like repeat domains of TNC regulated cell adhesion and cell motility and were anti-adhesion regions of fibroblasts, neurons, and glial cells. This domain also regulates neuronal migration and axon pathfinding during development. TNC has many isomers with different functions and sizes due to the alternative splicing of fibronectin type III (FNIII)-like repeats (Fig. 1) 11-22. Many studies have shown that TNC plays a key role in atherosclerosis. TNC expression was increased in the plaque in apoE-/- mice 23, 24. Serum TNC levels were increased in 307 patients with CHD 25-27 and 170 patients with ACS 28. The single nucleotide polymorphisms (SNPs) of TNC, such as rs3789875 and rs12347433, were related to atherosclerosis and CAD 29**.** TNC was rapidly upregulated after ischemic events, such as MI, suggesting that circulating TNC is a diagnostic or prognostic auxiliary biomarker in patients with cardiovascular disease 30. However, the friend and foe of TNC in atherosclerosis depended on its location *in vivo*. Interestingly, TNC was strongly overexpressed in atherosclerotic plaque active areas but was virtually undetectable in most normal adult tissues, especially the TNC C domain/isoform (TNC-C). G11, G11-iRGD, TN11, PL1, PL3, F16, and FNLM are promising delivery vectors for atherosclerosis-targeted drugs. In particular, G11-iRGD, PL1, and F16 have great development value because they contain or recognize components of clinical studies or their conjugate agents being investigated in clinical trials. In addition, to the best of our knowledge, ATN-RNA, a TNC-specific agent, was investigated in clinical trials as a therapeutic drug, while IMA950, a TNC agent, was investigated in clinical trials as a vaccine. Therefore, using TNC could greatly improve the success rate of atherosclerosis-targeted drugs and/or specific drug development. In this review, we focused on the potential of TNC in atherosclerosis, the delivery vector for atherosclerosis-targeted drugs, and TNC agents in the hope of providing knowledge for drug development by targeting TNC.

## 2. The role and mechanism of TNC in atherosclerosis

### 2.1 Anti-atherosclerotic effect and mechanism of TNC

TNC promoted coronary vessel development to primitive endothelial tubes by promoting the recruitment of α-smooth muscle actin (SMA)-positive mural cells to primitive endothelial tubes by stimulating the integrin αvβ3/PDGF-BB/PDGFRβ signaling pathway 24. TNC is also an endogenous ligand of TLR-4 and promotes chronic inflammatory and foam cell formation by activating the TLR-4-mediated NF-κB signaling pathway and CD36 expression in macrophages from THP-1 cells 31, 32. Macrophage TNC enhanced macrophage migration and VEGF release and activated the Akt/NF-κB and ERK pathways by binding to its receptor annexin II in RAW 264.7 macrophages and human primary macrophages 33. TNC promoted platelet adhesion, activation, and thrombosis by stimulating the integrin α(2)β(1) and α(IIb)β(3) by binding to Von Willebrand factor (VWF) 34. In addition, the TNC^+^ SMC subset was increased in plaques from human carotid and dog arteries. The TNC^+^ SMC subset promoted atherogenesis by enhancing ECM deposition and remodeling by increasing the expression of 11 related genes, including clusterin (CLU), collagen type XIV alpha 1 chain (COL14A1), ENSCAFG00000015206, fibrillin-1 (FBN1), LGALS3, matrix Gla protein (MGP), NADH dehydrogenase (ubiquinone) 1 alpha subcomplex 4-like 2 (NDUFA4L2), periostin (POSTN), serum amyloid A1 (SAA1), secreted modular calcium-binding protein 2 (SMOC2), and versican (VCAN) 35. Of note, the EGF-like domain of TNC promoted SMC apoptosis by activating caspase-3 22, 36. The A1 and A2 domains of TNC were mainly expressed in SMCs and promoted SMC chemotaxis and migration 22. The A1 and A2 domains of TNC also inhibited T-cell activation. The A2 domain of TNC suppressed the proliferation of human dermal microvascular endothelial cells. The fibrinogen-like globe (FBG) domain of TNC promoted IL-6, IL-8, and TNFα in human macrophages. The TnfnIII 1-5 domains of TNC inhibited aVb1 and a4b1-mediated adhesion to fibronectin 21. All of these factors were risk factors for atherosclerosis. Therefore, many TNC domains/isoforms promote atherosclerosis development, such as the A1 domain, A2 domain, EGF-like domain, FBG domain, and TnfnIII 1-5 domains. TNC from macrophages, platelets, and SMCs promoted atherosclerosis development.

### 2.2 Proatherogenic effect and mechanism of TNC

TNC not only stimulated pro-inflammatory cytokine expression, such as IL-6, IL-8, and TNFα but also anti-inflammatory cytokine expression, such as IL-4 and IL-13, suggesting that TNC has both pro-inflammatory and anti-inflammatory activity 37. However, knockout of TNC not only did not change the lipoprotein profile but also the adhesion, migration, and proliferation of SMCs 37, suggesting that the effect of TNC on atherosclerosis was independent of lipid metabolism and SMCs. Knockout of TNC also exacerbated the systemic inflammatory response and atherosclerosis by enhancing eotaxin (also named CCL11) levels in apoE^-/-^ mice 38. Interestingly, among the 62 inflammatory cytokines (such as Axl, CXCL16, IGFBP-3, IGFBP-6, IL-12 p70, Leptin R, LIX, soluble L-selectin, MIP-1γ, PF-4, soluble P-selectin, TNF-RI, TNFRII, and soluble VCAM-1), eotaxin was the only cytokine that was regulated by TNC, suggesting that eotaxin plays a key role in TNC-mediated atheroprotective activity 38. In another study, knockout of TNC exacerbated intraplaque hemorrhage and inflammation in endothelial cells (ECs) and macrophages to induce atherosclerosis development by enhancing VCAM-1 expression 37. Bone marrow-derived TNC reduced cardiac hypertrophy by reducing inflammation in mice 39. Thus, TNC from bone marrow and the whole body exhibited atheroprotective activity, while TNC from macrophages, platelets, and SMCs promoted atherosclerosis development.

## 3. The promising delivery vector for atherosclerosis-targeted drugs

### 3.1 G11

**Figure 2:**
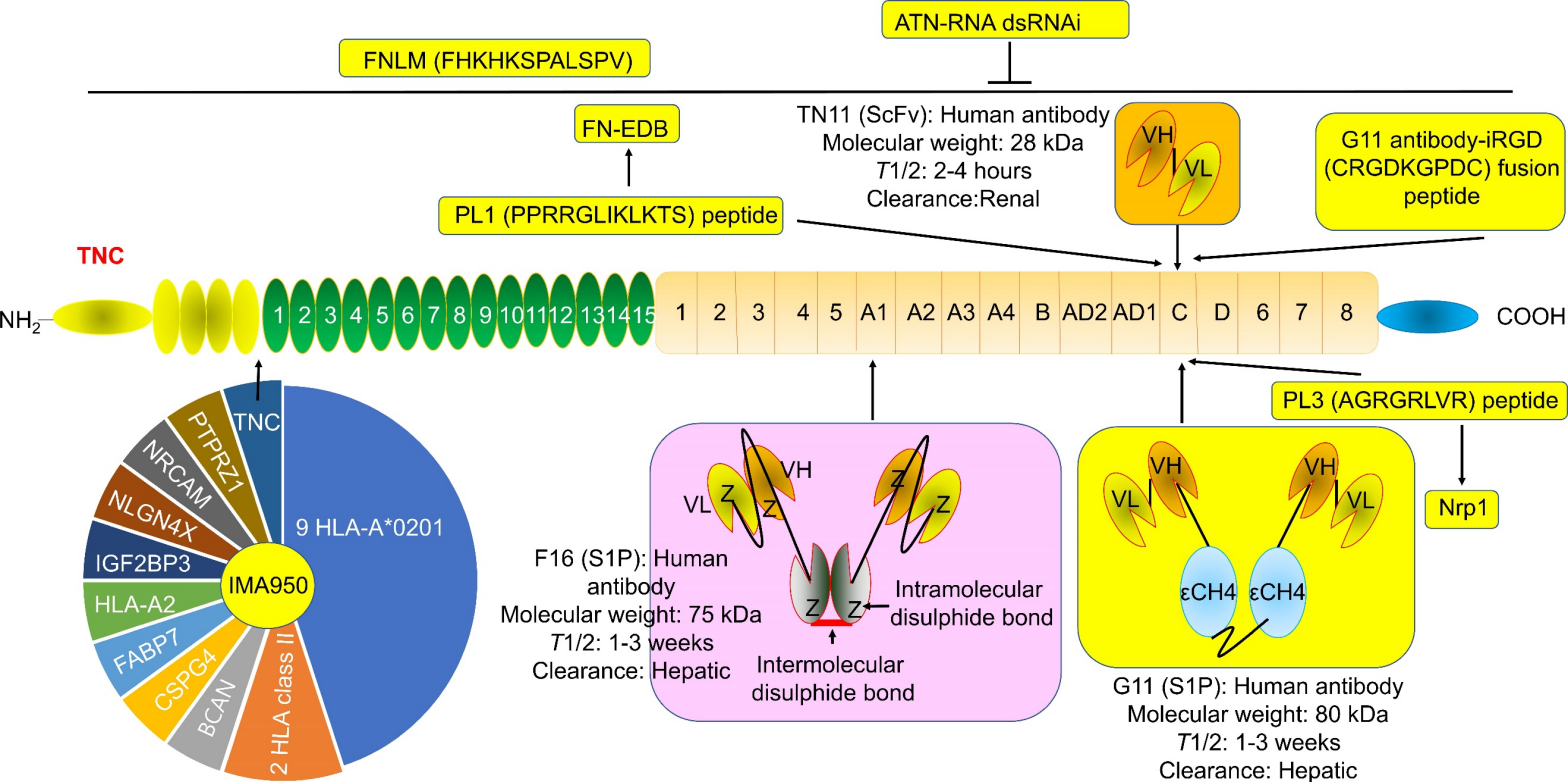
** The structure and location of TNC agents.** BCAN, bidirectional correct attention network; CSPG4, chondroitin sulfate proteoglycan 4; FABP7, fatty acid binding protein 7; FN-EDB, Extra Domain-B of fibronectin; IGF2BP3, insulin-like growth factor 2 mRNA binding protein 3; NLGN4X, neuroligin 4 X-linked; NRCAM, neuronal cell adhesion molecule; PTPRZ1, protein tyrosine phosphatase receptor type Z1; TNC, tenascin C; *T*_1/2_, half-life. The information was taken and modified from Refs 15, 40, 42, 46, 63, 65, 69, 74.

G11 (mini antibody SIP format) was a human antibody that only recognized TNC-C. G11 was used to observe the angiogenesis of advanced atherosclerotic plaques in mice, as well as gliomas and lung tumors 30. ^125^I (iodine)-G11 is a G11 with ^125^I labeling. ^125^I-G11 (intravenous injection, I.V., 0.3 MBq, 8 μg) was taken up in fat and activated macrophages in aortic plaques in apoE-/- mice. The sensitivity of autoradiography to detect plaque fatty stained areas was 74% and 76% at 4 hours and 24 hours after intravenous administration of ^125^I-G11. The signal-to-noise ratio (SNR) of vessel walls with plaque and without plaque was 43:1 and 104:1 at 4 hours and 24 hours, respectively. The SNR of activated macrophages in aortic plaques was 50:1 and 90:1 at 4 hours and 24 hours, respectively. The aorta-to-blood ratios of ^125^I-G11 were 0.45 and 1.53. After 4-24 hours of administration, the clearance of ^125^I-G11 was 86%, suggesting that the blood half-life of ^125^I-G11 was less than 20 min 30, 40. Therefore, G11 may facilitate molecular imaging of advanced atherosclerotic plaques and the delivery of related drugs. No G11 conjugate agents have been investigated in clinical trials. More studies are needed to confirm G11's development value. Of note, TNC-C was undetectable in normal adult tissue and exhibited a more restricted expression pattern than other TNC domains. TNC-C was also strongly abundant in macrophage-rich plaques but was not detected in normal adult tissues 30, 40, 41, suggesting that targeting TNC-C facilitated the development of diagnostic and targeting agents. Importantly, several other antibodies or antibodies can specifically recognize TNC-C, including G11-iRGD, TN11, PL1, and PL3 (Fig. 2).

G11-iRGD is a fusion peptide that contains the G11 antibody and iRGD peptide (sequence: CRGDKGPDC) 42. iRGD is also known as CEND-1 and LSTA1 and is a promising delivery vector. Interestingly, iRGD was investigated in clinical trials in cancer 43, 44. Therefore, G11-iRGD may be a codelivery system than G11 and iRGD. TN11 is a human antibody fragment antibody (scFV) that only recognizes TNC-C 45. However, to the best of our knowledge, the role of G11-iRGD, iRGD, and TN11 in atherosclerosis has not been investigated. No G11-iRGD or TN11 conjugate agents were investigated in clinical trials. More studies are needed to confirm the development value of G11-iRGD and TN11.

PL1 (sequence: PPRRGLIKLKTS), a peptide, recognizes not only TNC-c but also Extra Domain-B (EDB) of fibronectin (FN), which plays a key role in atherosclerosis development 46. However, FN is a double-edged sword in atherosclerosis. FN exhibited an anti-atherosclerotic effect by preventing plaque rupture and vascular occlusion by promoting the formation of thick fiber caps, while FN exhibited a proatherogenic effect by promoting lipoprotein retention and inflammatory cell infiltration by expanding the ECM 47-49. Interestingly, FN was also a promising delivery vector for atherosclerosis-targeted drugs, such as TPTS/C/T. TPTS/C/T is a simvastatin nanoprodrug (TPTS) with ROS-responsive cleavage properties and a fibronectin-targeted system. TPTS/C/T could codeliver the simvastatin prodrug and ticagrelor. TPTS/C/T exhibited stronger anti-inflammatory and antioxidant effects than free simvastatin by decreasing the M1-type polarization of macrophages, intracellular reactive oxygen species (ROS), and proinflammatory cytokines, such as IL-1β, MCP-1, and TNFα levels. In the apoE-/- mouse model of atherosclerosis, TPTS/C/T exhibited good synergistic therapy, anti-atherosclerosis effects, and biosafety by targeting the release of simvastatin and ticagrelor in plaques 50. In addition, L19 is a human recombinant antibody that specifically recognizes FN-EDB. L19 exhibited similar results with G11 in atherosclerotic plaques in apoE-/- mice 40. More importantly, many L19 antibody cytokine fusion proteins are currently being investigated in clinical trials, such as L19-IL2 (also named Darleukin) 51, 52, L19-TNF (also named Fibromun) 52, 53, and Nidlegy^TM^ (combination of L19-IL2 and L19-TNF) (Table 1) 52, 54. Several L19 antibody cytokine fusion proteins were also investigated in preclinical trials, such as L19-TNF-IL2 (also named Tripokin) 55, L19-IFNγ (also named L19-IFNγ KRG) 56, L19-IL12 57, and L19-IL15 58, 59. Therefore, PL1 may be a codelivery system other than G11, FN, and L19 because it specifically recognizes TNC-C and FN-EDB. However, the role of PL1 in atherosclerosis has not been investigated. No PL1 conjugate agents have been investigated in clinical trials. More studies are needed to confirm the development value of PL1.

PL3 (sequence: AGRGRLVR), a peptide, recognizes not only the C domain of TNC but also neuropilin-1 (Nrp1) 60. Nrp1, a type I transmembrane protein, is also a double-edged sword in atherosclerosis. Nrp1 from T cells promoted atherosclerosis development by promoting the recruitment of CD4 T cells into the aorta 61. Nrp1 suppressed leukocyte rolling and atherosclerotic plaque size by reducing proinflammatory cytokine and adhesion molecule levels by interacting with VE-cadherin and transforming growth factor-β (TGF-β) receptor II (TGFBR2) 62. In addition, the role of PL3 in atherosclerosis has not been investigated. No PL3 conjugate agents have been investigated in clinical trials. More studies are needed to confirm the development value of PL3.

### 3.2 F16

F16 is a fully human monoclonal antibody that only recognizes TNC A1 domain//isoform (TNC-A1) 63. F16 was investigated in atherosclerosis in clinical trials 64. F16 could bind to macrophages, blood vessels, and proliferating cells in human atherosclerotic plaque active areas but not to normal arteries and resting plaque areas (28 atherosclerotic plaques and 11 normal arteries), suggesting that the F16 antibody has strong specificity and selectivity for active plaques and may be a strong candidate for plaque imaging radiopharmaceuticals 64. Interestingly, the F16 antibody cytokine fusion protein F16IL2 (also named Teleukin) was investigated in clinical trials (Table 1). Therefore, F16 is a promising delivery vector for atherosclerosis-targeted drugs. However, in our capacity, we did not find any information about the F16 conjugate agents in atherosclerosis.

### 3.3 FNLM

FH (sequences, FHKHKSPALSPV) is a peptide that recognizes the large isoform of TNC. TNC is highly expressed in some inflammatory conditions, such as MI injury, but is sparsely expressed in normal adult myocardium. FNLM-miR consists of a hybrid membrane shell and MSNs-miR. The hybrid membrane shell is an artificial lipid membrane modified with FH peptide and fused with neutrophil membrane proteins (NMPs). MSNs-miR are mesoporous silica nanoparticles (MSNs) loaded with miR-1, miR-133a, miR-208, and miR-499 (miRCombo). FNLM-miR delivered more miRCombo into cardiac fibroblasts (CFs) in the injured heart to induce reprogramming into induced cardiomyocyte-like cells (iCMs). FNLM-miR reduced the expression of multiple cytokines, including CCR2, CXCR1, CXCR2, LFA-1, IL-1β, and IL-6. FNLM-miR also increased the expression of sarcomere-related genes, such as Myh6 and Tnnis, transcription factor-related genes, such as Gata4, Mef2c, and Tbx5, and ion channel-related genes, such as Kcnj2 and Scn5a. FNLM-miR (I.V.) induced reprogramming to improve cardiac function and alleviate fibrosis by delivering miRCombo into fibroblasts in a myocardial ischemia/reperfusion (MIR) injury mouse model. FNLM-miR has good safety, long blood circulation, and a fine fibroblast targeting profile in this model 65. These results suggest that FNLM-miR could deliver miRCombo into the cardiovascular system. FNLM is a promising delivery vector for atherosclerosis-targeted drugs. No FNLM conjugate agents have been investigated in clinical trials. More studies are needed to confirm the development value of FNLM.

## 4. Clinical advances in TNC-specific agents

### 4.1 ATN-RNA

ATN-RNA, an RNA interference (RNAi), is a double-stranded RNA (dsRNA) that targets TNC. ATN-RNA inhibited TNC synthesis *in vivo* 66, 67. ATN-RNA (injection into the brain after resection) for the treatment of brain tumors (N=46) inhibited tumor growth and disease recurrence. ATN-RNA prolonged the survival period of patients and improved the quality of life 67-69. The median overall survival (OS) of patients treated with ATN-RNA was 106.6 weeks and was higher than that of the control group (48.2 weeks). The longest survival was 180.9 weeks (Table 2) 69. These results support the continued development of ATN-RNA. However, the role of ATN-RNA in atherosclerosis was not investigated. Of note, the development of ATN-RNA has obtained patent protection, such as US8946400 (B2), EP2121927 (B1), US2010076053 (A1), EP2121927 (A2), WO2008016317 (A3), WO2008016317 (A2), and WO2008016317. However, since the last report of clinical results (2010), we have not found any clinical reports or clinical trials.

### 4.2 IMA950

IMA950, a multipeptide glioblastoma vaccine, contains 20 antigens from multiple protein-derived peptides, such as TNC, 9 HLA-A*0201-restricted peptides, 2 HLA class II (DR)-binding peptides (stimulated CD8^+^ and CD4^+^ T-cell response), HLA-A2-restricted peptides (immunopotency marker), bidirectional correct attention network (BCAN), chondroitin sulfate proteoglycan 4 (CSPG4), fatty acid binding protein 7 (FABP7), insulin-like growth factor 2 mRNA binding protein 3 (IGF2BP3), neuroligin 4 X-linked (NLGN4X), neuronal cell adhesion molecule (NRCAM), and protein tyrosine phosphatase receptor type Z1 (PTPRZ1) 70-75. IMA950 promoted the expression of BCAN, CSPG4, IGF2BP3, PTPRZ1, and TNC mRNA and protein in tumor samples from grade II and III glioma (49 grade II and 41 grade III astrocytoma, 30 grade II and 27 grade III oligodendroglioma, and 12 ependymoma). After vaccination, 100% of Grade II and 71% of Grade III patients had spontaneous antigen-specific T-cell responses. These patients showed a better T-cell response, suggesting that IMA950 improved the response to T-cell therapy 72. IMA950 in combination with granulocyte-macrophage colony-stimulating factor (GM-CSF) exhibited safety, tolerance, and immunogenicity in HLA-A*02-positive glioblastoma patients (N=45) in phase 1 clinical trials (intradermally). Ninety percent of patients (36/40) had tumor-associated peptides (TUMAP). Multi-TUMAP responders were 50%. The progression-free survival (PFS) at 6 months and 9 months was 74.4% and 30.8%, respectively. The disease stabilization rate at 40 weeks was 28.2% (11/39). The median OS was 15.3 months 73. IMA950 in combination with poly-ICLC (a synthetic TLR3 ligand) exhibited safety, tolerance, and immunogenicity in malignant astrocytoma patients (16 glioblastomas and 3 grade III astrocytoma) in phase 1/2 clinical trials (intradermally, intramuscularly, or subcutaneously). The median OS and disease control rates for all patients were 19 months (21 months after surgery) and 42%, respectively, including 17 months (19 months after surgery) and 31.2% of glioblastoma patients. The PFS for all patients and glioblastoma patients was 9 months and 9 months, respectively, while it was 10 months and 9.5 months after surgery, respectively. The mode of administration had no significant effect on OS and PFS 74. Bevacizumab (BEV), a monoclonal anti-VEGF-A IgG1 antibody, was approved for the treatment of multiple cancers, such as cervical cancer, colorectal cancer, glioblastoma, glioma, liver cancer, NSCLC, ovarian cancer, and renal cell carcinoma. However, IMA950/poly-ICLC did not improve the response rate, median OS, or median PFS of BEV for the treatment of relapsing high-grade glioma patients (vaccinated=16, nonvaccinated=40) 75. These novel results support the continued development of IMA950, except in combination with BEV. Indeed, many clinical trials on IMA950 are ongoing (Table 2). However, the further development of IMA950 in glioblastoma was discontinued on 23 September 2020 76.

## 5. Several issues of concern

TNC, especially TNC-C, is a promising target and delivery vector for drug development and atherosclerosis-targeted drugs. However, several issues of concern need to be noted. (1) Anti-TNC-USPIO, ultrasmall superparamagnetic iron oxide (USPIO) nanoparticle-labeled mouse anti-TNC monoclonal antibody, is a promising molecular imaging tool for detecting and monitoring atherosclerotic plaques by magnetic resonance imaging (MRI) in preclinical trials 23, 77. However, the role of anti-TNC-USPIO in clinical trials has not been investigated. (2) Many TNC domains/isoforms were investigated in atherosclerosis, such as EGF-like, A1, A2, FBG, C, and TnfnIII 1-5 domains. However, there are many domains/isoforms that have not been investigated. (3) Targeting TNC-C, such as G11, G11-iRGD, TN11, PL1, and PL3, is a promising delivery vector for atherosclerosis-targeted agents. However, no conjugate agents have been investigated in clinical trials. The role of G11-iRGD, TN11, PL1, and PL3 in atherosclerosis is also not unclear. (4) TTA1, a 13-kDa oligonucleotide (39-mer) aptamer, could recognize the TNC fibrinogen-like domain. TTA1 could target delivery to the ECM of atherosclerotic lesions and tumors 78. Therefore, TTA1 is a promising delivery vector for atherosclerosis-targeted agents.

However, no TTA1 conjugate agents have been investigated. The role of TTA1 in atherosclerosis is also not unclear. (5) IL-2 not only plays a key role in cancer but also in atherosclerosis 79-83. F16IL2 can deliver IL-2 to tumor tissue very well. F16 is a promising delivery vector for atherosclerosis-targeted agents. However, the role of F16IL2 in atherosclerosis has not been investigated. (6) Knockout and knockdown of TNC *in vivo* may suppress atherosclerosis development, suggesting that ATN-RNA may suppress atherosclerosis development by reducing TNC expression *in vivo*. However, the role of ATN-RNA in atherosclerosis has not been investigated. (7) The low uptake of ATN-RNA in cells weakens its anticancer ability and requires delivery vectors to enhance its uptake 84. Magnetic nanoparticles coated with polyethyleneimine (PEI) could improve the delivery of ATN-RNA in human U-118 MG cell lines 84, 85. However, the role of this nanomediated delivery of ATN-RNA *in vivo* is unclear. (8) TNC monoclonal antibodies may improve the delivery of ATN-RNA. However, attention should be given to cross-reactions within the TNC gene. (9) ATN-RNA also suppressed MDA-MB-231 breast cancer cell proliferation, migration, and adhesion *in vitro* 86. However, the role of ATN-RNA in suppressing breast cancer *in vivo* is unclear. (10) TNC is one of the antigens of the IMA950 vaccine. However, to the best of our knowledge, the role of TNC in IMA950 has not been investigated. (11) The investigation of the reasons for stopping the development of IMA950 is of great significance for restarting its development. Of note, GM-CSF and poly-ICLC were used as adjuvants for the IMA950 vaccine. However, these adjuvants are only ongoing in clinical research and are not found in marketed vaccines. Many adjuvants are approved for use in marketed vaccines, such as AS01 (TLR4 and NLRP3 agonist), AS03 (apoptosis-associated speck-like protein (ASC) agonist), AS04 (TLR4 and NLRP3 agonist), CpG ODN 1018 (TLR9 agonist), and MF59 (apoptosis-associated speck-like protein (ASC) agonist) 87-90. These adjuvants may be more suitable for the development of the IMA950 vaccine.

## 6. Conclusions

The pro-atherosclerosis or anti-atherosclerosis effect of TNC depended on its location. TNC was overexpressed in blood and atherosclerotic plaque active areas from patients with CHD. However, its expression is virtually undetectable in most normal adult tissues, especially TNC-C. Targeting TNC-C with G11, G11-iRGD, TN11, PL1, and PL3 is a promising delivery vector for atherosclerosis-targeted drugs. In particular, G11-iRGD and PL1 have great development value because they contain or recognize components of clinical studies. F16 and FNLM are also targeted drug delivery vectors with great development value due to their conjugate agents being investigated in preclinical and clinical trials, especially F16 (clinical trials). Targeting TNC is a promising target for drug development due to the specific agents ATN-RNA and IMA950 being investigated in clinical trials. With the deepening of research and cooperation in scientific research, it is believed that more scientists will develop more therapeutic (conjugate and/or specific agents), vaccine, and diagnostic agents using TNC as a delivery vector or target.

## Figures and Tables

**Table 1:** Clinical trials of iRGD, L19-IL2, L19-TNF, the combination of L19-IL2 and L19-TNF, and F16IL2.

Name	Combination	Disease	Status	Refs/ClinicalTrials
iRGD	Paclitaxel and gemcitabine	Advanced metastatic pancreatic ductal adenocarcinoma	Phase 1/2 (Recruiting on 13 January 2023)	NCT05052567
	FOLFIRINOX and panitumumab	Pancreatic, colon, and appendiceal cancers	Phase 1/2 (Recruiting on 24 November 2021)	NCT05121038
	Gemcitabine and nab-paclitaxel	Metastatic pancreatic cancer	Phase 1 (Completed on 6 July 2022)	43, 44
		Untreated metastatic pancreatic cancer	Phase 2 (Recruiting on 26 January 2023)	NCT05042128
	Dynamic contrast-enhanced magnetic resonance imaging (DCE-MRI)	Advanced breast or pancreatic cancer with metastases to the liver or lung	Phase 1 (Withdrawn on 30 May 2014)	NCT01741597
L19-IL2	Radiation	Non-Small Cell Lung Cancer	Phase 2 (Recruiting on 20 October 2020)	NCT03705403
L19-TNF	Doxorubicin	1st line Soft-Tissue Sarcoma	Phase 3 (Recruiting on 23 December 2022)	NCT04650984
	Doxorubicin	1st line Leiomyosarcoma	Phase 2 (Recruiting on 14 April 2022)	NCT03420014
	Dacarbazine	Pretreated Soft-Tissue Sarcoma	Phase 2 (Recruiting on 23 December 2022)	NCT04733183
	Lomustine	Glioblastoma	Phase 1 (Not yet recruiting on 20 April 2022)	NCT05304663
		Glioblastoma	Phase 1/2 (Recruiting on 22 June 2023)	NCT04573192
	Radiation and Temozolomide	Glioblastoma	Phase 1/2 (Recruiting on 14 April 2022)	NCT04443010
	Alone	Grade III/IV Glioma	Phase 1/2 (Completed on 29 June 2023)	NCT03779230
Combination of L19-IL2 and L19-TNF	Surgery	Stage III B/C Melanoma	Phase 3 (Recruiting on 11 April 2022)	NCT02938299
	Surgery and adjuvant therapy	Stage III B/C Melanoma	Phase 3 (Recruiting on 22 June 2023)	NCT03567889
	Alone	Basket of Non-Melanoma Skin Cancers	Phase 2 (Recruiting on 20 April 2022)	NCT04362722
	Alone	Skin Cancer	Phase 2 (Recruiting on 22 June 2023)	NCT05329792
F16IL2	Nivolumab	Non-small Cell Lung Cancer (NSCLC)	Phase 1/2 (Active, not recruiting on 21 July 2022)	NCT05468294
	Cytarabine	Acute Myeloid Leukemia (AML)	Phase 1/2 (Active, not recruiting on 20 April 2022)	NCT02957032
	Paclitaxel	Merkel Cell Carcinoma	Phase 1/2 (Terminated due to lack of enrollment on 18 May 2018)	NCT02054884
	BI 836858	AML Relapse	Phase 1 (Completed on 20 April 2022)	NCT03207191
	Doxorubicin	Solid Tumor	Phase 1/2 (Terminated on 25 February 2014)	NCT01131364
	Paclitaxel	Solid Tumor	Phase 1/2 (Completed on 15 April 2022)	NCT01134250

**Table 2:** ** The clinical trials and results of TNC agents ATN-RNA and IMA950.** GM-CSF, granulocyte-macrophage colony-stimulating factor; OS, overall survival; PFS, progression-free survival; TUMAP, tumor-associated peptide.

Name	Instructions	Diseases	Phase	Results					Developers	Refs
ATN-RNA	RNA interference	Brain tumors	Unknown	Toxicity	Safety				Institute of Bioorganic Chemistry of the Polish Academy of Sciences	69
				Efficacy	Median OS		106.6 weeks			
IMA950	Multipeptide vaccine	Glioblastoma	Unknown	Efficacy	Antigen-specific T-cell responses		Grade II glioma	100% (79/79)	Immatics Biotechnologies GmbH, Geneva University Hospital	72
							Grade III glioma	71% (48/68)		
		HLA-A*02-positive glioblastoma	Phase 1 (combination GM-CSF)	Toxicity	Safety				Cancer Research UK, Immatics Biotechnologies GmbH	73
				Efficacy	TUMAP		90% (36/40)			
					Median OS		15.3 months			
		Malignant astrocytoma	Phase 1 (combination poly-ICLC)	Toxicity	Safety				Immatics Biotechnologies GmbH, Geneva University Hospital	74
				Efficacy	Median OS (after surgery)		21 months			
					Disease control rate		42%			
					PFS (after surgery)		10 months			
		Relapsing high-grade glioma	Phase 1 (in combination with Bevacizumab and poly-ICLC)	IMA950/poly-ICLC did not improve the therapeutic effect of Bevacizumab (vaccinated=16, nonvaccinated=40)					Immatics Biotechnologies GmbH, Geneva University Hospital	75
	Pembrolizumab and Poly-ICLC	Relapsing Glioblastoma	Phase 1/2 (Active, not recruiting on 27 December 2022)						Geneva University Hospital	NCT03665545
	Varlilumab (also named CDX-1127) and Poly-ICLC	WHO Grade II Low-Grade Glioma (LGG)	Phase 1/2 (Active, not recruiting on 27 March 2023)						Nicholas Butowski, University of California, San Francisco, Celldex Therapeutics	NCT02924038
	Cyclophosphamide, Imiquimod, and GM-CSF	Glioblastoma	Phase 1 (Terminated due to poor accrual. on 19 May 2014)						Immatics Biotechnologies GmbH, National Cancer Institute	NCT01403285
	Temozolomide and Radiation Therapy	Newly Diagnosed Glioblastoma Multiforme	Phase 1 (Completed on 14 October 2015)			Unknown			Cancer Research UK, Immatics Biotechnologies GmbH	NCT01222221
										
